# Morintides: cargo-free chitin-binding peptides from *Moringa oleifera*

**DOI:** 10.1186/s12870-017-1014-6

**Published:** 2017-03-31

**Authors:** Shruthi G. Kini, Ka H. Wong, Wei Liang Tan, Tianshu Xiao, James P. Tam

**Affiliations:** School of Biological Sciences, Nanyang Technological University, Singapore, Singapore

**Keywords:** Cysteine-rich peptides, Hevein, Hevein-like peptides, *Moringa oleifera*, Anti-fungal, Chitin-binding

## Abstract

**Background:**

Hevein-like peptides are a family of cysteine-rich and chitin-binding peptides consisting of 29–45 amino acids. Their chitin-binding property is essential for plant defense against fungi. Based on the number of cysteine residues in their sequences, they are divided into three sub-families: 6C-, 8C- and 10C-hevein-like peptides. All three subfamilies contain a three-domain precursor comprising a signal peptide, a mature hevein-like peptide and a C-terminal domain comprising a hinge region with protein cargo in 8C- and 10C-hevein-like peptides.

**Results:**

Here we report the isolation and characterization of two novel 8C-hevein-like peptides, designated morintides (mO1 and mO2), from the drumstick tree *Moringa oleifera*, a drought-resistant tree belonging to the Moringaceae family. Proteomic analysis revealed that morintides comprise 44 amino acid residues and are rich in cysteine, glycine and hydrophilic amino acid residues such as asparagine and glutamine. Morintides are resistant to thermal and enzymatic degradation, able to bind to chitin and inhibit the growth of phyto-pathogenic fungi. Transcriptomic analysis showed that they contain a three-domain precursor comprising an endoplasmic reticulum (ER) signal sequence, a mature peptide domain and a C-terminal domain. A striking feature distinguishing morintides from other 8C-hevein-like peptides is a short and protein-cargo-free C-terminal domain. Previously, a similar protein-cargo-free C-terminal domain has been observed only in ginkgotides, the 8C-hevein-like peptides from a gymnosperm *Ginkgo biloba*. Thus, morintides, with a cargo-free C-terminal domain, are a stand-alone class of 8C-hevein-like peptides from angiosperms.

**Conclusions:**

Our results expand the existing library of hevein-like peptides and shed light on molecular diversity within the hevein-like peptide family. Our work also sheds light on the anti-fungal activity and stability of 8C-hevein-like peptides.

**Electronic supplementary material:**

The online version of this article (doi:10.1186/s12870-017-1014-6) contains supplementary material, which is available to authorized users.

## Background

Hevein, a 43-amino-acid-long-peptide comprising a conserved chitin-binding domain, was first isolated from the latex of the rubber tree *Hevea brasiliensis* by Archer et al. in 1960 [1]. Subsequently, peptides homologous to hevein were isolated from different plants and named hevein-like peptides due to their similarity to hevein. Hevein and hevein-like peptides, are a family of cysteine-rich peptides (CRPs), 29–45 amino acids in length, and are rich glycine (5–7 residues). Based on the number of cysteine residues, they can be classified into three subfamilies, namely 6C-, 8C- and 10C-hevein-like peptides [2] (Additional file 1: Table S1). Hevein-like peptides play a role in plant defense by binding to chitin, a polymer of repeating N-acetylglucosamine (GlcNAc) units and a major constituent of fungal cell walls [3]. Binding of hevein-like peptides to chitin is facilitated by a highly conserved domain called the hevein domain [4] or chitin-binding domain [5], which is composed of glycine, cysteine and aromatic residues stabilized by three to five disulfide bonds. The hevein domain is also found in chitin hydrolyzing enzymes called chitinases and *Urtica dioica* agglutinin (UDA) from stinging nettle *Urtica dioica* [6]. Due to their chitin-binding property, hevein-like peptides inhibit the growth of a wide range of fungi [7].

**Fig. 1:**
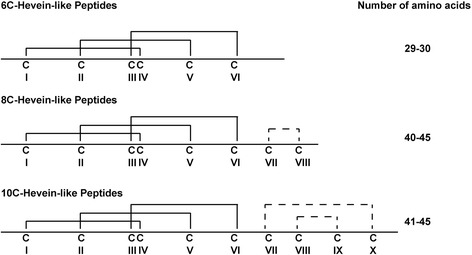
Comparison of disulfide patterns of 6C-, 8C- and 10C-hevein-like peptides. The conserved cystine knot motif is indicated in solid lines while the additional disulfides in 8C- and 10C-hevein-like peptides are indicated with dotted lines

Thus far, five 8C-hevein-like peptides, namely, hevein, two Fa-AMPs from *Fagopyrum esculentum* [8] and two Pn-AMPs from *Pharbitis nil* have been isolated from angiosperms [9]. Recently, our laboratory reported the discovery of eleven novel proline-rich, protein-cargo-free 8C-hevein-like peptides, named ginkgotides from the gymnosperm *Ginkgo biloba* [10]. The primary sequence of ginkgotides is distinctly different from angiospermic 8C-hevein-like peptides. The 8C-hevein-like peptides are 40–45 amino acids in length and contain four disulfide bonds. Three disulfide bonds at the N-terminus are arranged to form a cystine motif of Cys I-Cys IV, Cys II-Cys V, Cys III-Cys VI, whereas two C-terminal cysteines (Cys VII and Cys VIII) form the fourth disulfide bond. This fourth disulfide bond at the C-terminus is absent in 6C-hevein-like peptides [11, 12] whereas an additional disulfide bond in 10C-hevein-like peptides is present at the C-terminus [2] (Fig. 1).

**Fig. 2:**
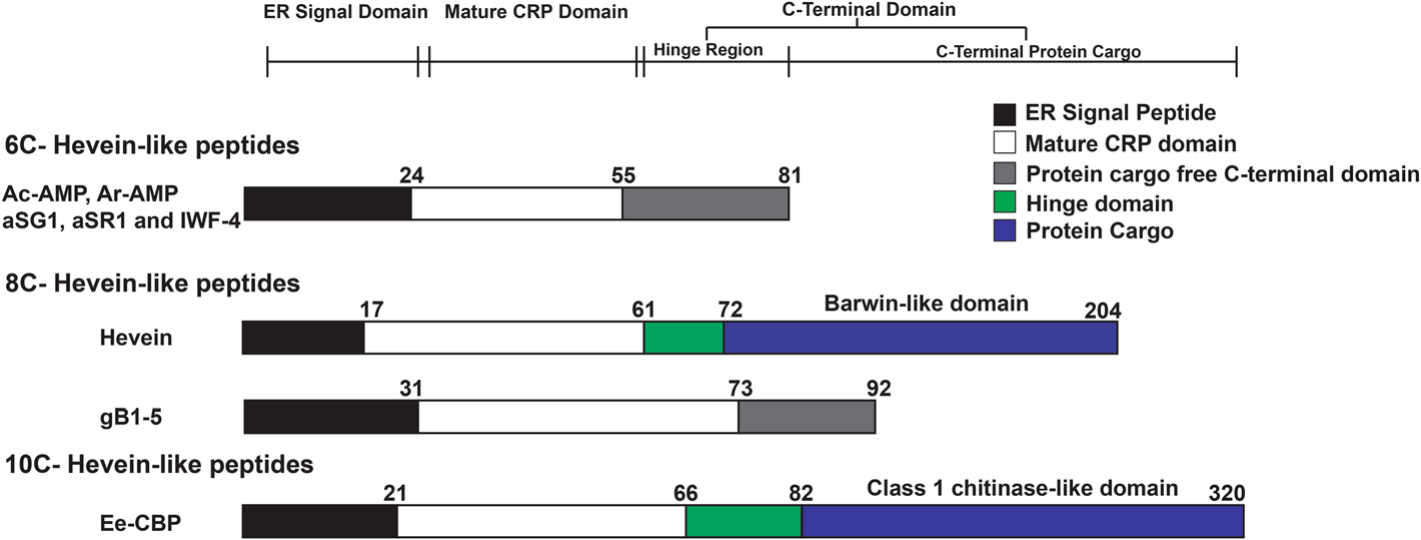
Schematic of precursor organization of hevein-like peptides. The precursors of hevein-like peptides comprise a signal peptide domain, a mature domain and a C-terminal domain. This precursor organization is conserved throughout the hevein-like peptide family. The C-terminal domain of hevein and Ee-CBP carry a bioactive protein cargo while 6C-hevein-like peptides have a protein-cargo-free C-terminal domain

Biosynthetic studies on 6C-, 8C- and 10C-hevein-like peptides showed that they share three-domain precursors consisted of a signal peptide domain, a mature peptide domain and a C-terminal domain [13]. Hevein-like peptide precursors can be hololectins, with a short C-terminal domain resembling the hinge domain of chitinases (~15-25 amino acids) or chimeric proteins where the C-terminal domain carries a protein cargo attached to the hinge region [14]. The 6C-hevein-like-peptides contain a short C-terminal domain of 25–30 residues, whereas the angiospermic 8C-hevein-like peptides contain a long C-terminal domain that usually encodes for bioactive protein cargo, such as proteins with a Barwin-like domain [13, 15, 16]. However, in ginkgotides, isolated from gymnosperms, the C-terminal domain is short and protein cargo-free. Similarly, 10C-hevein-like peptides, including Ee-CBP from *Euonymus europaeus,* contain a long C-terminal domain carrying a class-I-chitinase-like protein cargo [14] (Fig. 2) whereas the C-terminal domain of WAMPs isolated from *Triticum kiharae* comprise a short, protein-cargo-free C-terminal domain [17].

*Moringa oleifera* belongs to the flowering plant family Moringaceae and is commonly known as the drumstick tree or the horse-radish tree [18, 19]. It was once native to the foothills of the Himalayas but is now cultivated globally, especially in tropical and sub-tropical areas [20, 21]. It is a drought-resistant tree that can grow to a height of 10 m, and its trunk can reach a diameter of 45 cm [22, 23]. *M. oleifera* leaves are a rich source of vitamin C, calcium, potassium, β-carotene and natural antioxidants [18]. The stem-bark, flowers, seeds and roots are edible and nutritious, comprising compounds such as alkaloids, potassium, calcium, ascorbic acid and antioxidants [24, 25]. Since *M. oleifera* is rich in nutrients, it is commonly referred to as the “Miracle Tree” to combat malnutrition in developing countries [26]. Due to its high medicinal and nutritional value, *M. oleifera* is now incorporated into health formulations that are marketed as remedies for a variety of health disorders [27]. *M. oleifera* seeds are the best known natural coagulants and are commonly used to purify turbid water. The coagulation properties are likely due to dimeric proteins in the seeds that are highly stable and soluble in water [28]. Aqueous and ethanol extracts of *M. oleifera* leaves are known to have anti-inflammatory, antimicrobial, anti-diabetic and anti-ulcer properties and cholesterol lowering and blood pressure stabilizing effects [29]. The seeds, stem, roots and leaves of *M. oleifera* are reported to exhibit anti-fungal effects [18, 29]. However, there is no report on the peptide and protein components that contribute to the putative therapeutic value of *M. oleifera* leaves. Since hevein-like peptides are primarily involved in plant defense against fungi, we screened the leaves of *M. oleifera* for putative hevein-like peptides by mass spectrometric analysis.

In this study, we report two antifungal 8C-hevein-like peptides from *M. oleifera*, designated morintides and abbreviated as mO1 and mO2. Sequencing using LC-ESI-MS/MS and transcriptomic analysis showed that morintides mO1 and mO2 are 44 amino acids long. Transcriptome data (RNAseq) analysis revealed that the full-length precursors of morintides comprise, a signal peptide domain, a mature peptide domain and a short C-terminal domain. Interestingly, morintides have a protein-cargo-free C-terminal domain (15 residues) when compared to hevein (142 residues). Since mO1 was the most abundant peptide, its function and stability were characterized. As a consequence of its chitin-binding property, mO1 inhibited the growth of the fungal strains *Alternaria alternata* and *Alternaria brassiciola,* by inhibiting the growth of fungal hyphae. To the best of our knowledge, this is the first report of an 8C-hevein-like peptide from angiosperms without a C-terminal protein cargo. Together, our results provide insight into the sequence diversity of hevein-like peptides, with insights into their chitin-binding property, anti-fungal activity and high stability against proteolysis and thermal degradation.

## Methods

### General experimental procedures

Shimadzu systems were used for high-performance liquid chromatography (HPLC) and ultra-performance liquid chromatography (UPLC). Phenomenex C18 columns (particle size, 5 μm; pore size, 300 Å; CA, USA) with dimensions of 250 × 22 mm, 250 × 10 mm, and 250 × 4.6 mm were used for preparative, semi-preparative, and analytical reverse-phase (RP)-HPLC, respectively. Strong cation exchange (SCX)-HPLC was performed on a polyLC polysulfoethyl A column (250 × 9.4 mm and 250 × 4.6 mm). Mass spectrometry (MS) analysis of crude extracts and HPLC fractions was performed using an ABI 4800 MALDI-TOF/TOF system (Applied Biosystems, Framingham, MA, USA). An Infinite® 200 PRO microplate reader (Tecan Group Ltd., Maennedorf, Switzerland Germany) was used to measure absorbance in chitin binding, cytotoxicity, and microbroth dilution assays. Chemical reagents of analytical grade were purchased from Sigma Aldrich (St. Louis, MO, USA) [30–33].

### Isolation of peptides from *M. oleifera*

*M. oleifera* leaves (3 kg) were bought from a local market in Singapore, and were authenticated by an experienced herbalist Mr Ng Kim Chuan from Nanyang Herbs Garden (Nanyang Technological University, Singapore). A voucher specimen was deposited in the Nanyang Technological University Herbarium, School of Biological Sciences, Singapore. The fresh leaves were blended with an equal volume of water in an Oster Fusion Blender (Philips, Singapore) for 6 min. The homogenized plant material was then centrifuged at 8,000 rpm for 10 min at 4 °C in an Avanti J25 centrifuge (Beckman-Coulter, USA) [34, 35]. The supernatant was filtered and loaded onto a C18 flash column. Elution was performed using increasing concentrations of ethanol (20 ­ 70%). The presence of the desired peptides was confirmed by performing MALDI-TOF MS on the eluents. Fractions that contained the peptides of interest were then pooled and concentrated using a rotary evaporator at a pressure of 70 mbar at 42 °C. The concentrated eluents were then purified using multiple rounds of SCX and RP-HPLC. For SCX-HPLC a gradient from buffer A (20 mM NaH_2_PO_4_, 5% acetonitrile, pH 2.8) to buffer B (20 mM NaH_2_PO_4,_ 5% acetonitrile, 0.5 M NaCl, pH 2.8) was used. Fractions from SCX-HPLC that contained the desired peptides were pooled and purified by RP-HPLC using a linear gradient from buffer A (0.1% trifluoroacetic acid in water) to buffer B (0.1% trifluoroacetic acid in 100% acetonitrile).

### RNA extraction

Extraction of RNA from fresh *M. oleifera* leaves was performed using TRIzol® Reagent (Life Technologies, Carlsbad, USA) following the manufacturer’s protocol. Briefly, fresh *M. oleifera* leaves (100 mg) were homogenized using liquid nitrogen and incubated with 1 ml of TRIzol® Reagent (Life Technologies, Carlsbad, USA) at room temperature for 5 min. Chloroform (200 μL) was added to the homogenized sample and incubated at room temperature for 3 min followed by centrifugation at 12,000 g for 15 min at 4 °C. The RNA-containing aqueous phase was incubated at room temperature for 10 min with an equal volume of isopropanol before centrifuging at 12,000 g for 10 min. The pellet containing RNA was washed twice with 75% ethanol (1 mL). The pellet was dried and resuspended in 30 μl of diethylpyrocarbonate (DEPC) water. The RNA sample was sent to Macrogen Inc. Korea for sequencing.

### Sequence determination of morintides

Reduction and alkylation of morintides was performed as previously described [15, 36, 37]. Briefly, approximately 600 μg of purified peptide was incubated with 20 mM dithiothreitol (DTT) in 20 mM ammonium bicarbonate buffer (NH_4_HCO_3_, pH 7) at 37 °C for 2 h. The reduced peptide was then alkylated with 40 mM iodoacetamide at 37 °C for 1 h. The reduced-alkylated sample was desalted using Millipore ZipTips and lyophilized. The peptide was re-dissolved in 0.1% formic acid before mass spectrometry analysis. A Dionex UltiMate 3000 UHPLC system (Thermo Fisher Scientific, Bremen, Germany) coupled with an Orbitrap Elite mass spectrometer (Thermo Scientific Inc., Bremen, Germany) was used to perform LC/MS-MS analysis. Elution was performed over a 60 min gradient from eluent A (0.1% formic acid) to eluent B (90% acetonitrile/0.1% formic acid). LTQ Tune Plus software (Thermo Fisher Scientific, Bremen, Germany) was used to set the mass spectrometer to positive mode for data acquisition. A Michrom’s Thermo CaptiveSpray nanoelectrospray ion source (Bruker-Michrom, Auburn, USA) was used to generate the spray. A Full FT-MS (350–2000 m/z, resolution 60.000, with 1 μscan per spectrum) was alternated with Full FT-MS and an FT-MS/MS scan applying 27%, 30 and 32% normalized collision energy in high-energy collisional dissociation (110–2000 m/z, resolution 30,000, with 2 μscan averaged per MS/MS spectrum) for data acquisition where three intense ions with a charge greater than +2 and a mass difference of 3 Da were isolated and fragmented. A source voltage of 1.5 kV and capillary temperature of 250 °C were used. Automatic gain control was set to 1 × 10^6^ for full scan-MS and MS/MS. PEAKS studio (version 7.5, Bioinformatics Solutions, Waterloo, Canada) was used to process data from LC-MS/MS analysis with parent error tolerance and a fragment error tolerance of 10 ppm and 0.05 Da, respectively.

### Phylogenetic analysis

The precursor sequences were aligned using MUSCLE [38] and analyzed using neighbor joining agglomerative clustering by MEGA 6.0 [39]. The phylogenetic tree was constructed using bootstrap method with 1000 replications and poisson model was employed for substitution modelling. The phylogenetic tree was displayed using iTOL [40].

### Chitin binding assay

Purified morintide (mO1) was incubated with chitin beads (New England Biolabs, Massachusetts, UK) in chitin binding buffer (140 mM NaCl, 10 mM Tris EDTA 1 mM; pH 8.0; then, 0.1% (v/v) Tween 20 was added) at 4 °C for 4 h. At each time point, the beads were centrifuged at 12,000 g for 1 min and the absorbance of the supernatant was read at 280 nm to assess binding. Elution of the bound peptide was performed using two methods. In the first method, 0.5 M acetic acid and heating at 100 °C for 30 min and 1 h was used, and in the second method, four elution buffers (10 mM Tris, 1 mM EDTA, 0.1% (v/v) Tween 20; pH 8.0) with increasing concentration of sodium chloride (300 mM, 500 mM, 700 mM, and 1 M) were used. Reduced and alkylated mO1 was used as a control. Supernatants were further analyzed by UPLC and MALDI-TOF to assess binding and elution.

### Disc diffusion assay

The susceptibility of seven fungal strains obtained from the China Center of Industrial Culture Collection (CICC), namely, *Curvularia lunata* (CICC 40301), *Fusarium oxysporum* (CICC 2532), *Aspergillus niger* (CICC 2089), *Verticillium dahilae* (CICC 2534), *Rhizoctonia solani* (CICC 40259), *Alternaria alternata* (CICC 2465) and *Alternaria brassiciola* (CICC 2646) to mO1 (17.5 to 70 μg) was tested using a disc diffusion assay [41]. Potato dextrose agar (PDA) plates were used to grow *C. lunata, R. solani, V. dahilae A. alternata*, and *A. brassiciola* (*A. niger* was grown on malt extract (ME) agar plates) at 25 °C. When sufficient growth was observed, the mycelia were harvested by punching a hole in the growing fungi and transferring it to a new agar plate. The plate was incubated at 25 °C for 48 h to 72 h to allow formation of a radial mycelial colony. 6 mm discs impregnated with 20 μL of mO1 were placed equidistant (1 cm) from the growing ends of the mycelia and incubated for 24 h at 25 °C. Deionized water was used as a negative control. Formation of arc-shaped inhibition zones indicated susceptibility to mO1.

### Microbroth dilution assay

A half maximal inhibitory concentration (IC_50_) of mO1 against *A. alternata* and *A. brassiciola* was obtained by performing a microbroth dilution assay as described by Wiegand et al. [42]. Fungal spores were seeded in half-strength potato dextrose broth at a density of 2.5 × 10^3^ cells/mL. Peptide samples (900 μg/mL to 0.05 μg/mL) were added to the spore suspensions in a 96-well plate and incubated for 24 h at 25 °C. After incubation, the cells were fixed with 100% methanol for 30 min followed by staining with 1% (w/v) methylene blue in 0.01 M borate buffer for 30 min [43]. Excess dye was washed with water, the plates were dried, and stain was eluted with 1% (v/v) ethanol/0.1 N HCl. Absorbance was read at 650 nm using an Infinite@ 200 PRO Tecan microplate reader (Tecan Group Ltd, Germany). Control microcultures contained half-strength potato dextrose broth alone. Percentage growth inhibition was calculated as 100 times the ratio of absorbance of the test microcultures over the absorbance of the control microcultures.

### Thermal stability assay

Purified mO1 (40 μg) was dissolved in water and incubated at 100 °C for 1 h. A linear peptide RV-14 (RLYRRGRLYRRNHV) synthesized in our laboratory was used as a control. After incubation, the samples were subjected to RP-UPLC to assess the presence and extent of degradation.

### Proteolytic enzyme stability assays

Purified mO1 was incubated at 37 °C with pepsin or carboxypeptidase A in 100 mM sodium citrate (pH 4.5) or 50 mM Tris–HCl with 100 mM NaCl (pH 7.5) at a final peptide to enzyme ratio (w/w) of 20:1 and 50:1, respectively. At each time point (0, 2, 4, 6 h) 50 μL of the sample was injected into the UPLC system, and the mass of peaks collected were analyzed by MALDI-TOF MS. A linear peptide WV-14 (WRLYRGRLYRRNHV) synthesized in-house was used as a control.

### Cytotoxicity assay

The cytotoxic effect of morintide, mO1 (1–100 μM) was tested on Vero cells (5 × 10^4^ cells/ml) in Dulbecco’s modified Eagle’s medium (DMEM) supplemented with 10% fetal bovine serum (FBS) and 1% penicillin/streptomycin (PS). Vero cells (ATCC CCL-81) were a kind gift from Dr Liu Ding Xiang (School of Biological Sciences, Nanyang Technological University, Singapore). After 24 h of incubation, 10 μl of PrestoBlue® Cell Viability Reagent (Invitrogen, USA) was added to the wells and incubated at 37 °C for 1 h. After incubation, the absorbance was measured at 570 nm using a microplate reader.

### NMR experiments and structure determination

The assignment and structure determination were achieved using 2D ^1^H-^1^H TOCSY and NOESY experiments. The spectra were recorded using a Bruker 800 MHz NMR spectrometer (Bruker, IL, USA) equipped with a cryogenic probe. The temperature for the NMR experiments was 298 K. The concentration of mO1 peptide was 1 mM. The lyophilized peptide was dissolved in Milli-Q water, containing 5% D_2_O (pH approximately 3.5). The mixing times of TOCSY and NOESY experiments were 80 and 200 ms, respectively. The center of the spectrum was 4.735 ppm, and the spectrum width was 12 ppm. The spectra were processed using the software NMRpipe [44]. Assignment of the NOESY spectrum was accomplished using the software Sparky 3.115 [45]. To identify the amide protons involved in hydrogen bonds, an H/D exchange NMR experiment was conducted at 298 K, using a Bruker 600 MHz NMR spectrometer (Bruker, IL, USA) equipped with a cryogenic probe. The lyophilized peptide was dissolved in 100% D_2_O. 1D NMR spectra were recorded a long time after the peptide dissolved in D_2_O. In total, 13 spectra were recorded for 12 h. The structure calculation was done using the software CNSsolve 1.3 [46]. The structures were verified by the server PDBsum [47] to generate a Ramachandran plot. The structure is displayed by the software Chimera 1.6.2 [48].

## Results

### Screening and isolation of peptides from *M. oleifera*

A small-scale screening of *M. oleifera* leaves revealed a cluster of peptides, with masses of approximately 4 kDa. A scale-up extraction of 3 kg of plant material was performed by mincing in an equal volume of water. Putative cysteine-rich peptides (CRPs) were isolated by multiple rounds of SCX and RP-HPLC. The MS profile of *M. oleifera* indicated that two major peptide peaks with m/z of 4536.71 Da and 4463.76 Da were present in the leaf extract and were designated morintide mO1 and mO2, respectively. After reduction and alkylation of the disulfide bonds, a mass shift of +474 Da was observed indicating the presence of eight cysteine residues. Sequencing of morintides was performed by nanospray MS/MS. Overall, the sequence contained cysteine (eight residues), glycine (six residues), asparagine (five residues) and glutamine (five residues), making up approximately 50% of the morintide peptide sequence. The glutamine residue at the N-terminus was spontaneously converted to pyroglutamic acid, making the peptide resistant to degradation by exopeptidases.

Since tandem MS cannot differentiate isobaric residues, such as Leu/Ile, they were assigned based on the transcriptome data obtained from RNA extracted from fresh *M. oleifera* leaves. The primary sequence of morintides differed in one amino acid position: Gln-15 in mO1 was replaced by Gly-15 in mO2 (Table 1). Sequence alignment revealed a cysteine-spacing pattern of X_2_C-X_8_C-X_4_CC-X_5_C-X_6_C–X_5_C–X_3_C–X_3_ with an absolutely conserved chitin binding domain SX*Φ*X*Φ*CGX_4_ *Φ* in loops 3 and 4, where X represents any amino acid and *Φ* represents Trp, Tyr or Phe. BLAST analysis and a conserved domain search on NCBI revealed that morintides belonged to the hevein-like peptide family, shared greater than 54% sequence identity with 8C-hevein-like peptides and the highest sequence identity (64.3%) with hevein and the least sequence identity (42.9%) with ginkgotides from *G. biloba*.

**Table 1:**
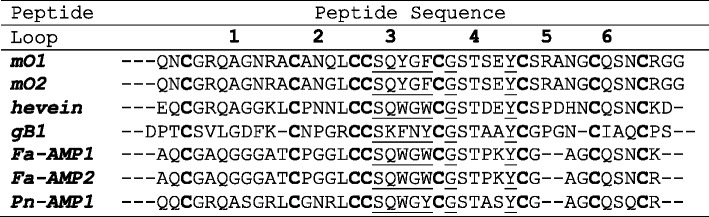
Comparison of morintide sequences with other 8C-hevein-like peptides

Comparison with 8C-hevein-like peptides [1, 8, 9] showed that morintides shared highest sequence identity with hevein and all peptides contained a conserved chitin-binding domain in loops 3 and 4 (underlined).

### NMR structure of morintide mO1

**Fig. 3:**
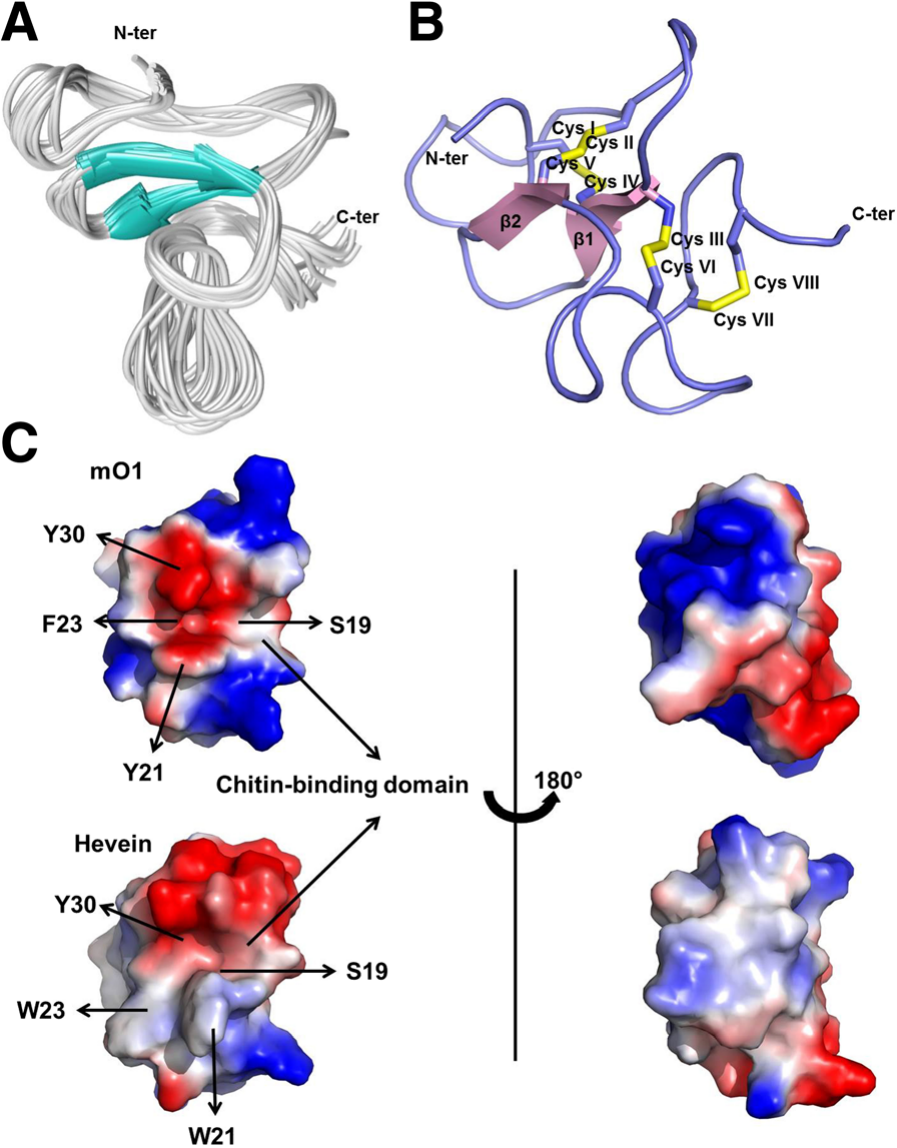
NMR structure of mO1. **a** Superposition of the backbone traces from 20 ensemble structures and restrained energy minimized structures (REM) of mO1. **b** Ribbon representation of NMR structure of mO1. The disulfide bonds are indicated in yellow and β-sheets in pink. **c** Surface topology comparison of mO1 and hevein

The sequential assignment of mO1 was performed based on the NOE cross peaks between Hα(i-1), Hβ(i-1) and HN(i). Each amide proton HN should have NOE cross peaks with the side protons of the previous residue. Accordingly, the peaks in the NOESY spectrum were assigned without ambiguity. The structures generated by CNSsolve 1.3 are convergent. The backbone RMSD is 1.01 ± 0.35 Å for 41 residues (Q1-C41), and the heavy atom RMSD is 1.71 ± 0.33 Å (Additional file 2: Table S2). As shown in Fig. 3, mO1 contains two anti-parallel β strands similar to other hevein-like-peptides, with the strand β1 covering Cys-17 to Ser-19 and β2 covering Phe-23 to Gly-25. To confirm the disulfide bond connectivity of mO1, the averaged overall energies of the 20 best structures generated by CNSsolve 1.3 were compared among the different combinations of disulfide bonds. In the NOESY spectrum, the cross peak between the H_β_s of Cys-37 and Cys-41 was observed, indicating the close distance between the two H_β_s. Moreover, these two H_β_s had no cross peaks with the H_β_s of the other cysteines, further validating the disulfide bond between Cys-37 and Cys-41 (Cys VII- Cys VIII). The disulfide bond pattern for the remaining six cysteine residues were identified by generating 15 different combinations of disulfide bonds assuming that each one in the simulated annealing procedure generated 100 structures with the same distance restraints (Additional file 3: Table S3 and Additional file 4: Table S4). For each combination, the 20 structures with the lowest overall energy were selected, of which the averaged overall energy is listed in Additional file 2: Table S2. The combination (Cys I-Cys IV, Cys II-Cys V, Cys III-Cys VI) exhibiting the lowest averaged overall energy (618.67 ± 7.54 kcal/mol), indicating the correct disulfide bond pattern, was Cys I-Cys IV, Cys II-Cys V, Cys III-Cys VI, and Cys VII-Cys VIII. In addition, we assumed that all the cysteines were reduced for the structure calculation. Assuming there are no disulfide bonds the averaged overall energy became even lower (Additional file 3: Table S3). This is attributed to the absence of the structural restraints due to disulfide bonds. The structures generated in this case were almost the same as the ones with the disulfide pattern, Cys I-Cys IV, Cys II-Cys V, Cys III-Cys VI, Cys VII-Cys VIII, confirming that the deduced disulfide pattern is correct (Additional file 5: Figure S1). The four disulfide bonds tie the loops to the β strands. The disulfide bond Cys I-Cys IV and Cys II-Cys V cross and tie loop 1 and loop 2 to β1 and β2. The disulfide bond Cys III-Cys VI makes ties loop 4 to β1. The disulfide bond Cys VII-Cys VIII is located at the C terminus, bending the C terminus like a clamp. The four disulfide bonds maintain the structure of the peptide and thus are responsible for the high stability of mO1. In the H/D exchange experiment, the signals of the amide protons of S19, Q38, C17, C18, C24 and S39 decayed less than 20% after 12 h, indicating a high stability of mO1. A comparison of the chitin-binding domain and the distribution of charges on the surface of hevein and mO1 is shown in Fig. 3c. From the figure, it is observed that Tyr-30 is absolutely conserved, whereas positions 21 and 23 are occupied by Tyr and Phe, respectively. On comparison with the chitin-binding domain of hevein it was observed that the topography of the surface electric charge of hevein is relatively neutral, the corresponding region in mO1 seems to be surrounded by negatively charged residues (Fig. 3c).

### Chitin binding activity

To assess the chitin binding activity of morintides, mO1 was incubated with chitin beads for 4 h at 4 °C. RP-UPLC analysis showed that mO1 bound to chitin beads within 1 h (Additional file 6: Figure S2). Using elution buffers with up to 1 M NaCl, mO1 could not be eluted from the beads indicating a strong binding interaction. Use of 0.5 M acetic acid with heating at 100 °C effectively eluted mO1 in 1 h (Additional file 6: Figure S2). These data indicate that mO1 binds strongly to chitin beads, as it only could be eluted using a strong elution buffer and heating at high temperature.

### Anti-fungal activity of morintide

**Fig. 4:**
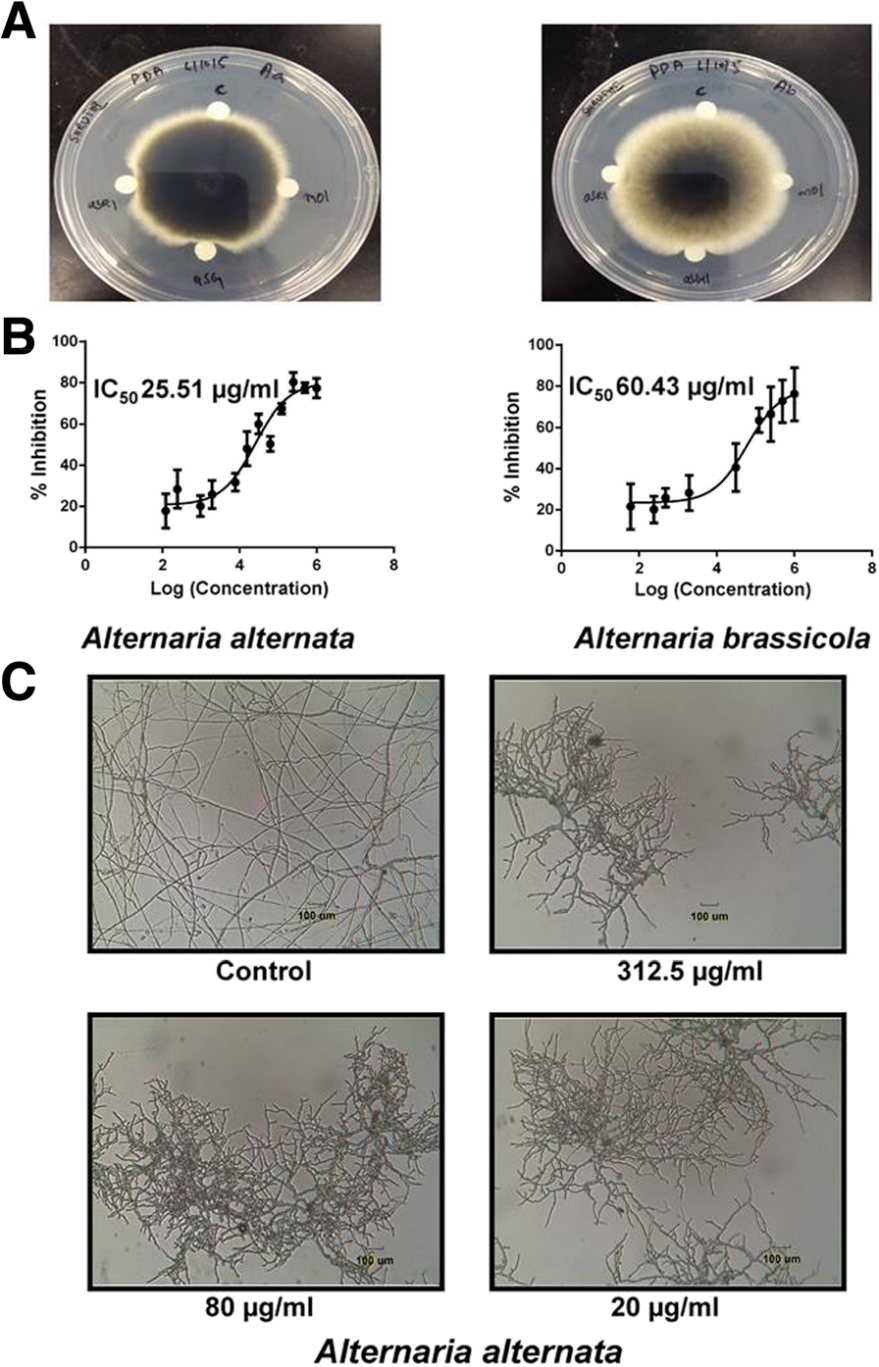
**a** Formation of arc-shaped inhibition zones at the growing tips of fungal mycelia indicates the susceptibility of *A. alternata* and *A. brassicola* to mO1. **b** Dose–response curves generated from the microbroth dilution assay were used to calculate the IC_50_ values. **c** Hyphal growth inhibition of *A. alternata* spores treated with different concentrations of mO1. Fungi treated with mO1 show distinct morphological changes. The hyphae are stunted, thick and less-branched when compared to the untreated control

A disc diffusion assay was performed to test the vulnerability of seven strains of phyto-pathogenic fungi to mO1. Fungi were inoculated and allowed to grow at 25 °C for 24–72 h or until a radial colony was formed. Morintides of designated concentrations were loaded on discs placed at the growing mycelial tips. Out of the seven strains *A. alternata* and *A. brassiciola* showed the formation of crescent-shaped inhibition zones indicating that they are susceptible to treatment with mO1 (Fig. 4a). A dose–response curve was generated from which the IC_50_ calculated was in the range of 25.5 μg/ml to 60.43 μg/ml after incubation for 24 h at 25 °C depending on the fungal strain tested (Fig. 4b). Bright field microscopy showed that treatment of fungal spores with mO1 resulted in morphological changes, such as short and thick hyphae when compared to the untreated control. The mO1-treated fungal mycelia were closely bunched together and did not branch out as extensively as the untreated fungi (Fig. 4c). Together, these data suggest that mO1 inhibits fungal growth by retarding the growth of budding hyphae in a dose-dependent manner.

### Heat and proteolytic stability

**Fig. 5:**
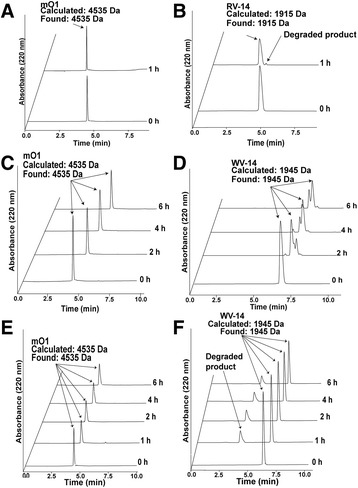
Stability assays of mO1. Thermal stability assay of (**a**) mO1 and (**b**) control peptide RV-14 (RLYRRGRLYRRNHV). **c** Carboxypeptidase stability assay of mO1 (**d**) Control peptide WV-14 (WRLYRGRLYRRNHV) degraded within 6 h upon treatment with carboxypeptidase. **e** Pepsin stability assay of mO1 (**f**) Control peptide WV-14 degraded within 6 h of pepsin treatment

Stability of mO1 against thermal and enzymatic degradation was assessed by incubating mO1 at 100 °C for 1 h or with pepsin and carboxypeptidase A for 6 h. By computing the area under the curve from RP-UPLC chromatograms, it was observed that more than 95% of the peptide remained intact after heating for 1 h and on treatment with proteolytic enzymes (Fig. 5). More than 50% of linear control peptide RV-14 was degraded within 1 h of heating at 100 °C while WV-14 was completely digested in 4 h by carboxypeptidase A, and only 50% of WV-14 remained intact after 6 h of pepsin treatment.

### Biological activity

The cytotoxic effect of morintide was tested on Vero cells using a PrestoBlue cell viability reagent. No significant cytotoxic effect was observed with concentrations up to 100 μM.

### Biosynthesis of morintides

A total of 78,561 transcripts with contig length between 201–22191 bp were assembled from transcriptome of RNA extracted from fresh *M. oleifera* leaves using Trinity after Illumina HiSeq performed by Macrogen Inc., Korea. These transcripts were analyzed using in-house software, Protein Analyzer 1.0 to look for conserved cysteine motifs. Two precursor sequences that were designated *mo1* and *mo2* were obtained. The precursors *mo1* and *mo2* comprise a 20-amino-acid-long ER signal peptide followed by a 44-amino acid long mature peptide domain and a short 15 amino acid-long C-terminal domain. Sequence comparison of *mo1* and *mo2* revealed that the signal peptide is identical, the mature domain differs in one amino acid residue, and the C-terminal domain varies at three positions, specifically, Asp-67, Glu-71 and Gly-76 of *mo1* are replaced by Ala-67, Gly-71 and Ser-76 in *mo2*. The precursor organization of hevein-like peptides differs from other cysteine-rich peptides that contain an N-terminal pro-domain before the mature peptide domain [30, 49, 50] and suggests that morintides are ribosomally synthesized peptides [51] whose bioprocessing occurs through the secretory pathway [52].

**Fig. 6:**
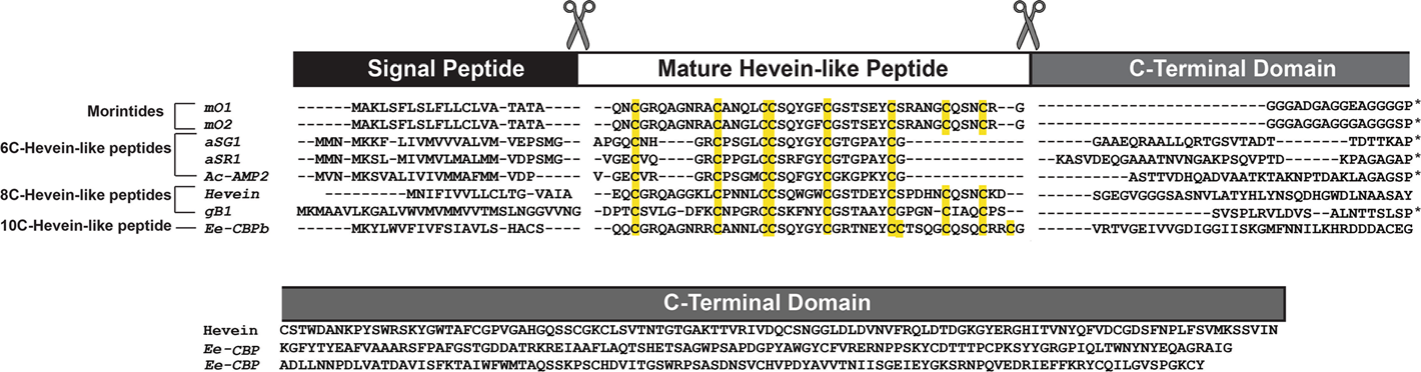
Sequence alignment of precursors of hevein-like peptides. The overall precursor organization of hevein-like peptides is conserved and comprises a signal peptide, mature hevein-like peptide and a C-terminal domain. The length of the C-terminal domain is highly variable throughout the hevein-like peptide family. Morintides comprise a short C-terminal domain similar to gB1, a ginkgotide from G. biloba. This precursor organization is similar to 6C-hevein-like peptides and differs from hevein and Ee-CBP that comprise long C-terminal domains that encode for bioactive protein cargo

The morintide precursor sequences were aligned with other hevein-like peptides, including Ac-AMP2 from *Amaranthus caudatus* [11], aSG1 from *Alternanthera sessilis* var. green [15], aSR1 from *Alternanthera sessilis* var. red [15], hevein from *Hevea brasiliensis* [16], gB1 from *Ginkgo biloba* and Ee-CBP from *Euonymus europaeus* [14] (Fig. 6). From Fig. 6, it is clear that the overall precursor organization of morintides is similar to ginkgotides, 8C-hevein-like peptides from the gymnosperm *G. biloba* [10].

## Discussion

In this study, two novel 8C-hevein-like peptides, collectively named morintides, were isolated, sequenced and characterized The 44 amino-acid-long morintides, were detected at proteomic and transcriptomic levels and differ by one amino acid residue. Morintides are homologous to hevein and other 8C-hevein-like peptides such as Pn-AMPs [9] and Fa-AMPs [8]. Structural analysis of mO1 by NMR revealed that three disulfide bonds were arranged to form the cystine knot motif, and the fourth disulfide bond was located at the C-terminus. Morintide mO1 inhibited the growth of *A. alternata* and *A. brassiciola* in the disc diffusion assay with an IC_50_ in the micromolar range. Transcriptomic analysis revealed that the overall precursor organization of morintides is similar to hevein-like peptides except for their short, protein-cargo-free C-terminal domain.

### Sequence comparison of morintides

Sequence comparison of morintides with hevein and reported 8C-hevein-like peptides revealed that, in addition to the eight conserved cysteine residues, three glutamine (Gln-6, Gln-20, Gln-36), two serine (Ser-19, Ser-37) and Leu-16 were absolutely conserved in all 8C-hevein-like peptides (Table 1). Based on the cysteine-spacing pattern, the peptide sequences can be divided into six intercysteinyl loops. The length of the loops is conserved, with variation observed only in loop 5 of Fa-AMPs and Pn-AMPs that comprises three residues as opposed to the five residues seen in hevein and mO1. The N-terminus of all 8C-hevein-like peptides, except Fa-AMPs, contains a glutamate residue that is spontaneously converted to pyroglutamic acid. The C-terminus usually consists of a positively charged residue followed by glycine, serine or aspartate residues in mO1, Pn-AMP1 and hevein, respectively. Morintides displayed the least sequence identity with the proline-rich ginkgotides, probably because they are isolated from a gymnosperm, *G. biloba*.

**Fig. 7:**
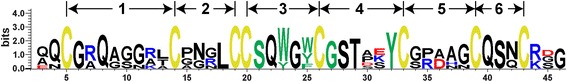
Sequence logo of morintides and nine reported hevein-like peptides. Sequence variability among 8C-hevein-like peptides is high as none of the loops are absolutely conserved. The chitin-binding domain is highlighted in green and is found in loops 3 and 4

A sequence logo of the aligned morintides and 8C-hevein-like peptides was used to illustrate the similarity and frequency of occurrence of amino acid residues at each position (Fig. 7). From the sequence logo it is clear that the extent of molecular diversity is high among 8C-hevein-like peptides. Loops 3 and 4 make up the chitin-binding domain where a serine and glutamine are absolutely conserved. The aromatic amino acids in loop 3 are variable, whereas a tyrosine residue in loop 4 is absolutely conserved. From these observations it is clear that despite sequence variations important residues involved in binding to chitin are well conserved, indicating the universality in diversity of 8C-hevein-like peptides.

### Morintide structure

The overall structure of CRPs is stabilized primarily by the disulfide-rich core along with the secondary structure and hydrophobic contacts [53]. The disulfide core of hevein-like peptides has a knotted topology characterized by two adjacent disulfide bonds that are crossed-over by a third disulfide bond resulting in the cystine-knot confirmation [54]. Consequently hevein-like peptides are highly stable to degradation by heat and enzymes. Morintide, mO1 has a similar disulfide arrangement. The secondary structure of hevein-like peptides, including hevein itself [55], Ac-AMP2 from *Amaranthus caudatus* [56], aSG1 from *Alternanthera sessilis* [15], WAMP-1a from *Triticum kiharae* [57] and Ee-CBP from *Euonymus europaeus* [58], comprises at least two β-strands and a short $\alpha$-helical turn. The secondary structure of mO1 is similar to the reported hevein-like peptides and comprises two anti-parallel β-sheets but lacks the $\alpha$-helical turn at the C-terminus. The C-terminal residue of mO1 is Arg, as opposed to Pro-33, which is found in hevein. This replacement could result in the absence of the $\alpha$-helical turn in mO1. The chitin-binding property of hevein-like peptides is determined by the residues occupying the chitin-binding site located close to the knottin core. In hevein, binding interactions with the N-acetylglucosamine residues are stabilized by CH-π stacking interactions and van der Waal’s contacts involving Trp-21, Trp-23 and Tyr-30 [59]. A hydrogen bond between the sugar moiety and the hydroxyl group of Ser-19 stabilizes the complex. Due to sequence homology of hevein and mO1 it is presumed that the chitin-binding interaction is through a similar interaction in mO1.

### Anti-fungal activity of morintides

Of the seven fungal strains tested, the morintide mO1 could inhibit the growth of *A. alternata* and *A. brassiciola,* as indicated by the arc-shaped inhibition zones in the disc diffusion assay. Morphological changes, such as swollen hyphal tips, stunted growth and less branched hyphae, were observed after treatment of *A. alternata* and *A. brassiciola* with different concentrations of mO1. Similar changes in morphology have been observed with hevein [7], IWF-4 [60] and Ee-CBP [58, 61]. At present, there are two proposed mechanisms by which hevein-like peptides may inhibit fungal growth. First, the chitin-binding property of hevein-like peptides might cause them to bind to nascent chitin fibers in newly formed chitin chains of growing hyphae, disrupt cell wall morphogenesis and impede fungal growth [60]. Second, the small size and highly basic PI of hevein-like peptides might allow them to easily penetrate through the fungal cell wall, reach the plasma membrane, and alter membrane polarity, causing leakage of cytoplasmic material and subsequently inhibiting fungal growth [9, 58, 61]. The mechanism of action of morintides remains to be elucidated; however, the anti-fungal activity of morintides can be attributed to their chitin-binding property as they have neutral PI values.

### Biosynthesis of morintides

Transcriptome data analysis revealed that morintides are expressed as three-domain precursors and are processed via the secretory pathway [52]. The N-terminal cleavage site of the signal peptidase is a highly conserved Ala residue in morintides, while C-terminal processing occurs at an absolutely conserved Gly residue (Additional file 7). The 15-amino-acid-long C-terminal domain of morintides is rich in glycine residues and differs from hevein and 10C-hevein-like peptides, which have a significantly longer C-terminal domain (142–200 amino acid residues) carrying a bio-active protein cargo with a Barwin domain or a glycine or serine-rich hinge region followed by the catalytic domain of class-I chitinases [62, 63]. To determine whether the C-terminal domain of mo1 plays a role as a targeting signal in directing the mature peptide to vacuoles, a blastp search was performed in the NCBI database. The search result shows no homology to any known proteins in the NCBI database. Thus the role of the C-tail in morintides remains to be determined.

The glycine-rich nature of the C-terminal domain of morintides suggests that it could be a remnant of the hinge-region in chitinases, and morintides could be truncated chitinases. The precursor organization of hevein-like peptides is similar to ginkgotides [10] and thionins [64] but differs from other CRPs such as cyclotides, which contain an N-terminal pro-peptide before the mature peptide [31, 36, 49, 65].

### Evolution and origin of morintides

The hevein-like peptide family can be divided into three sub-classes, making it an interesting family to study genetic diversity. Genetic divergence within the plant phyla results from mutations in the mature peptide, which also lead to functional diversification. For example, the signal peptide and C-terminal domain of morintide mO1 and mO2 precursors are highly homologous, but a point mutation in the mature domain results in replacement of Gln-15 in mO1 to Gly-15 in mO2. Similar genetic diversity has also been observed in other CRPs such as cliotides cT4-cT12 of the cyclotide family isolated from *Clitoria ternatea* [49], allotides of the cystine knot α-amylase family isolated from *Allamanda cathartica* [50] and α- and β-hordothionin of the thionin family isolated from *Hordeum vulgare* [66].

**Fig. 8:**
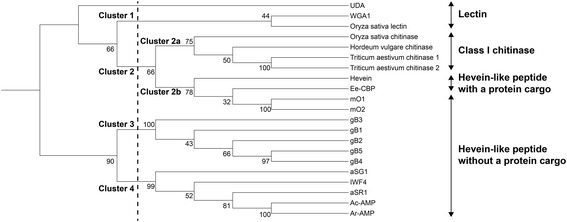
Phylogenetic tree of hevein-like peptides, chitinases and lectins. Morintides and hevein-like peptides are clustered separately probably due to the distinct nature of their C-terminal domain. Morintides are clustered close to chitinases suggesting there is a close evolutionary relationship between them and that the C-terminal domain of morintides might be a remnant of chitinases

A phylogenetic tree was constructed using the precursor sequences of morintides, reported hevein-like peptides, class I chitinases and lectins to study their evolutionary relationship (Fig. 8). Data analysis showed four distinct clusters as indicated by the dashed line in Fig. 8. Morintides are grouped in the same cluster (Cluster 2b) as hevein and Ee-CBP, which are adjacent to chitinases (Cluster 2a) suggesting that there is a close evolutionary relationship between hevein-like peptides and chitinases. This clustering further suggests that the C-terminal domain of morintides could be a remnant of the chitinase domain, through frame-shift deletions or mRNA splicing events. Our analysis is in agreement with Andreev et al., who speculated that wamp genes were “remnants” of chitinase genes. These “truncated” genes were then selected by evolution as they coded for anti-fungal peptides [17]. Our analysis showed that morintides, which belong to angiosperm, and gingkotides, which belong to gymnosperm, are located in different clusters, consistent with their evolutionary origins [10].

## Conclusion

In this study, two novel 8C-hevein-like peptides (mO1 and mO2), collectively named morintides, were isolated and characterized from the leaves of *M. oleifera*. NMR studies of the 44-amino-acid-long morintides revealed that they contain four disulfide bonds. The highly disulfide-constrained structure renders mO1 resistant to thermal and enzymatic degradation, making it an interesting scaffold for drug development. Morintide, mO1 can inhibit the growth of *A. alternata* and *A. brassiciola* with IC_50_ in the micromolar range. It caused morphological changes such as swollen hyphal tips with stunted and less-branched hyphae. Transcriptome data analysis revealed that morintide precursors comprise a signal peptide domain, a mature peptide domain and a C-terminal domain. Comparison of morintide precursors with other hevein-like peptides showed that they contain a radically shortened C-terminal domain, which could be the result of mRNA splicing or frame shift deletions in large chitinase precursors. This stark difference in the length of the C-terminal domain is an interesting finding that sheds light on the evolution of hevein-like peptides and helps define a novel sub-group of 8C-hevein-like peptides. Together, this work expands the library of 8C-hevein-like peptides and sheds light on their evolutionary relationship and biosynthetic pathway. We have also gained insights into the chitin-binding property and the fungal growth inhibition of morintides.

## Additional files

Additional file 1: Table S1. Aligned sequences of reported hevein-like peptides. (DOCX 23 kb)

Additional file 2: Table S2. Statistics of the structure of mO1 generated by CNSsolve 1.3. (DOCX 16 kb)

Additional file 3: Table S3. The comparison between the averaged overall energies of the different combinations of disulfide bonds. (DOCX 17 kb)

Additional file 4: Table S4. Chemical shift list of mO1 (DOCX 21 kb)

Additional file 5: Figure S1. The structures generated by CNSsolve 1.3 without any disulfide bonds assumed for structure calculation (A) and with the disulfide bonds combination Cys I- Cys IV, Cys II-Cys V, Cys III- Cys VI and Cys VII- Cys VII assumed for structure calculation (B) (JPG 28 kb)

Additional file 6: Figure S2. Chitin-binding activity of mO1. Morintide mO1 bound to the chitin beads in 1 h and was eluted from the beads in 30 min on incubation in acidic conditions at high temperature. (JPG 357 kb)

Additional file 7: Translated nucleotide sequences of mO1 and mO2 as obtained from transcriptomic analysis. An asterisk indicates the start codon and a hyphen indicates the stop codon. (DOCX 90 kb)

## Abbreviations

Ac-AMP: *Amaranthus caudatus*-AMP
AMP: Antimicrobial peptide
COSY: Correlation spectroscopy
CRP: Cysteine-rich peptide
D_2_O: Deuterium oxide
EDTA: Ethylenediaminetetraacetic acid
ER: Endoplasmic reticulum
GlcNAc: N-acetylglucosamine
HPLC: High-performance liquid chromatography
MALDI-TOF MS: Matrix-assisted laser desorption/ionization-time of flight mass spectrometry
NOESY: Nuclear Overhauser effect spectroscopy
PCR: Polymerase chain reaction
RP: Reversed-phase
SCX: Strong cation exchange
UPLC: Ultra-performance liquid chromatography
