# Supplementary material for: Morintides: cargo-free chitin-binding peptides from Moringa oleifera
Source: BMC Plant Biol. 2017 Mar 31;17:68. doi: 10.1186/s12870-017-1014-6 (PMC5374622; doi:10.1186/s12870-017-1014-6)
Supplement: Supplementary file 1 — Aligned sequences of reported hevein-like peptides. (DOCX 23 kb) [file 12870_2017_1014_MOESM1_ESM.docx]

Table S1. Aligned sequences of reported hevein-like peptides.

| **Peptide** | **Peptide Sequence** |
| --- | --- |
|  | *** * ** * * * * *** |
| *aSG1* | AP-GQ**C**NH----GR**C**PSGL**CC**SQYGY**C**GTGPAY**C**G-------------  **6C-HLPs** |
| *aSR1* | -V-GE**C**VQ----GR**C**PPGL**CC**SRFGY**C**GTGPAY**C**G------------- |
| *Ac-AMP2* | -V-GE**C**VR----GR**C**PSGM**CC**SQFGY**C**GKGPKY**C**G------------- |
| *Hevein* | ---EQ**C**GRQAGGKL**C**PNNL**CC**SQWGW**C**GSTDEY**C**SPDHN**C**QSN**C**KD--  **8C-HLPs** |
| *gB1* | --DPT**C**SVLGDFK-**C**NPGR**CC**SKFNY**C**GSTAAY**C**GPGN-**C**IAQ**C**PS-- |
| *Pn-AMP1* | ---QQ**C**GSQARGRL**C**GNGL**CC**SQWGY**C**GSTAAY**C**G--AG**C**QSQ**C**KS-- |
| *Ee-CBP* | ---QQ**C**GRQAGNRR**C**ANNL**CC**SQYGY**C**GRTNEY**CC**TSQG**C**QSQ**C**RR**C**G  **10C-HLPs** |
| *WAMP-1b* | --AQR**C**GDQARGAK**C**PN**C**L**CC**GKYGF**C**GSGDAY**C**G-AGS**C**QSQ**C**RG**C**R |

Hevein-like peptides (HLPs) can be divided into three sub-families, 6C-, 8C- and 10C-hevein-like peptides based on their cysteine content. Cysteine residues are indicated by an asterisk. The reported hevein-like peptides include aSG1 and aSR1 from *A. sessilis*, hevein from *H. brasiliensis*, gB1 from *G. biloba*, Pn-AMP1 from *P. nil*, Ee-CBP from *E. europaeus* and WAMP-1b from *T. kiharae.*
