# Supplementary material for: Morintides: cargo-free chitin-binding peptides from Moringa oleifera
Source: BMC Plant Biol. 2017 Mar 31;17:68. doi: 10.1186/s12870-017-1014-6 (PMC5374622; doi:10.1186/s12870-017-1014-6)
Supplement: Supplementary file 2 — Statistics of the structure of mO1 generated by CNSsolve 1.3. (DOCX 16 kb) [file 12870_2017_1014_MOESM2_ESM.docx]

Table S2. The comparison between the averaged overall energies of the different combinations of disulfide bonds.

|  | Disulfide Bond Pattern | Energy (kcal/mol) |
| --- | --- | --- |
| 0 | No Disulfide Bond Imposed | 581.85±9.19 |
| 1 | CysI-CysIV, CysII-CysIII, CysV-CysVI, CysVII-CysVIII | 840.83±9.84 |
| 2 | CysI-CysIV, CysIII-CysV, CysII-CysVI, CysVII-CysVIII | 803.81±9.55 |
| 3 | CysI-CysII, CysIII-CysIV, CysV-CysVI, CysVII-CysVIII | 957.13±12.33 |
| 4 | CysI-CysII, CysIII-CysVI, CysIV-CysV, CysVII-CysVIII | 672.80±6.08 |
| 5 | CysI-CysII, CysIV-CysVI, CysIII-CysV, CysVII-CysVIII | 799.88±21.08 |
| 6 | CysI-CysIII, CysII-CysIV, CysV-CysVI, CysVII-CysVIII | 968.86±14.31 |
| 7 | CysI-CysIII, CysII-CysV, CysIV-CysVI, CysVII-CysVIII | 828.75±12.49 |
| 8 | CysI-CysIII, CysII-CysVI, CysV-CysIV, CysVII-CysVIII | 957.53±12.72 |
| 9 | CysI-CysV, CysII-CysIII, CysIV-CysVI, CysVII-CysVIII | 777.26±9.64 |
| 10 | CysI-CysV, CysII-CysIV, CysIII-CysVI, CysVII-CysVIII | 653.27±9.98 |
| 11 | CysI-CysV, CysII-CysVI, CysIV-CysIII, CysVII-CysVIII | 1087.52±14.69 |
| 12 | CysI-CysVI, CysII-CysIII, CysIV-CysV, CysVII-CysVIII | 1210.01±87.84 |
| 13 | CysI-CysVI, CysII-CysV, CysIII-CysIV, CysVII-CysVIII | 1176.47±15.35 |
| 14 | CysI-CysVI, CysII-CysIV, CysV-CysIII, CysVII-CysVIII | 1059.67±10.59 |
| 15 | CysI-CysIV, CysII-CysV, CysIII-CysVI, CysVII-CysVIII | 618.67±7.54 |
