# Supplementary material for: Morintides: cargo-free chitin-binding peptides from Moringa oleifera
Source: BMC Plant Biol. 2017 Mar 31;17:68. doi: 10.1186/s12870-017-1014-6 (PMC5374622; doi:10.1186/s12870-017-1014-6)
Supplement: Supplementary file 3 — The comparison between the averaged overall energies of the different combinations of disulfide bonds. (DOCX 17 kb) [file 12870_2017_1014_MOESM3_ESM.docx]

| Experimental Restraints and Structural Statistics of 20 Lowest-Energy Structures of mO1 among the 100 Structures Generated by CNSsolve 1.3 |
| --- |
| NMR Distance Restraints |
| Intra-Residue NOE 144 |
| Sequential NOE 120 |
| Medium-Range NOE 83 |
| Long-Range NOE 92 |
| Hydrogen Bonds 4 |
|  |
| Structural Statistics (43 residues, Q^1^-G^43^) |
| Violations per Structure |
| NOE Violation (Å) 0.029±0.002 |
| Maximum NOE Violation(Å) 0.031 |
|  |
| Ramachandran Plot Region (43 residues) |
| Residues in Most Favored Regions 13 36.1% |
| Residues in Additional Allowed Regions 21 58.3% |
| Residues in Generously Allowed Regions 2 5.6% |
| Residues in Disallowed Regions 0 0% |
| Number of End-Residues (excl. Gly and Pro) 1 |
| Number of Glycine Residues 6 |
| Number of Proline Residues 0 |
|  |
| Mean RMSD from the Average Coordinates (41 residues, Q^1^-C^41^) |
| Backbone Atoms(Å) 1.01±0.35 |
| Heavy Atoms(Å) 1.71±0.33 |
|  |

Table S3. Statistics of the structure of mO1 generated by CNSsolve 1.3*.*
