# Supplementary material for: Morintides: cargo-free chitin-binding peptides from Moringa oleifera
Source: BMC Plant Biol. 2017 Mar 31;17:68. doi: 10.1186/s12870-017-1014-6 (PMC5374622; doi:10.1186/s12870-017-1014-6)
Supplement: Supplementary file 4 — Chemical shift list of mO1 (DOCX 21 kb) [file 12870_2017_1014_MOESM4_ESM.docx]

Table S4. Chemical shift list of mO1

| ResID | HN (ppm) | Hα (ppm) | | | Hβ (ppm) | | | Others (ppm) | |
| --- | --- | --- | --- | --- | --- | --- | --- | --- | --- |
| Q1 |  | 4.385 |  | 1.654 | |  | HΥ, 2.294 | |  |
| N2 | 8.538 | 4.68 |  | 2.829 | | 2.719 |  | |  |
| C3 | 8.136 | 4.762 |  | 2.998 | | 2.719 |  | |  |
| G4 | 8.303 | 3.861 | 3.661 |  | |  |  | |  |
| R5 | 9.549 | 4.048 |  | 1.821 | | 1.745 | HΥ, 1.665, 1.39 | |  |
| Q6 | 9.017 | 4.075 |  | 1.975 | | 1.943 | HΥ, 2.671, 2.37, Hδ, 7.616, 6.577 | |  |
| A7 | 7.774 | 4.528 |  | 0.748 | |  |  | |  |
| G8 | 8.278 | 3.736 | 3.669 |  | |  |  | |  |
| N9 | 8.243 | 3.661 |  | 2.938 | | 2.885 |  | |  |
| R10 | 6.997 | 4.142 |  | 1.788 | |  | HΥ, 1.722, Hδ, 3.219, 3.165 | |  |
| A11 | 8.69 | 4.419 |  | 1.393 | |  |  | |  |
| C12 | 8.875 | 4.399 |  | 2.921 | |  |  | |  |
| A13 | 8.619 | 4.28 |  | 1.368 | |  |  | |  |
| N14 | 8.656 | 4.321 |  | 2.888 | | 2.754 |  | |  |
| Q15 | 8.342 | 3.717 |  | 2.146 | |  | HΥ, 2.25, 2.209, Hδ, 7.616, 6.577 | |  |
| L16 | 7.162 | 4.271 |  | 1.71 | |  | HΥ, 1.467, Hδ, 0.772, 0.662 | |  |
| C17 | 8.343 | 4.592 |  | 4.095 | | 2.707 |  | |  |
| C18 | 8.045 | 5.212 |  | 3.026 | | 2.984 |  | |  |
| S19 | 9.88 | 4.901 |  | 4.338 | | 4.248 |  | |  |
| Y21 | 7.33 | 4.579 |  | 3.618 | | 2.728 | Hδ, 7.153, 7.245 | |  |
| G22 | 7.915 | 3.863 | 3.54 |  | |  |  | |  |
| F23 | 7.398 | 5.237 |  | 3.412 | | 2.929 | Hδ, 7.14, 6.722 | |  |
| C24 | 8.942 | 5.743 |  | 2.936 | | 2.861 |  | |  |
| G25 | 9.246 | 3.705 | 2.051 |  | |  |  | |  |
| S26 | 8.655 | 5.025 |  | 3.98 | | 3.622 |  | |  |
| T27 | 7.041 | 4.849 |  | 4.793 | |  | HΥ, 1.342 | |  |
| S28 | 9.32 | 4.182 |  | 3.966 | |  |  | |  |
| E29 | 8.371 | 4.066 |  | 1.95 | | 1.875 | HΥ, 2.309, 2.263 | |  |
| Y30 | 7.747 | 3.877 |  | 3.124 | | 2.805 | Hδ, 7.483 | |  |
| C31 | 7.665 | 4.78 |  | 3.163 | | 2.766 |  | |  |
| S32 | 7.911 | 4.711 |  | 4.055 | | 3.919 |  | |  |
| R33 | 10.048 | 4.051 |  | 1.898 | | 1.817 | HΥ, 1.682, Hδ, 3.196, Hε, 7.237 | |  |
| A34 | 8.689 | 4.167 |  | 1.34 | |  |  | |  |
| N35 | 7.283 | 5.004 |  | 3.15 | | 2.517 |  | |  |
| G36 | 7.892 | 3.935 | 3.738 |  | |  |  | |  |
| C37 | 7.162 | 4.257 |  | 2.042 | | 3.243 |  | |  |
| Q38 | 9.541 | 4.468 |  | 1.707 | |  | HΥ, 2.246, 2.149 | |  |
| S39 | 7.487 | 4.462 |  | 4.046 | | 3.766 |  | |  |
| N40 | 9.086 | 4.274 |  | 2.917 | | 2.811 |  | |  |
| C41 | 7.948 | 4.763 |  | 3.547 | | 2.509 |  | |  |
| R42 | 8.426 | 4.574 |  | 1.922 | |  | HΥ, 1.677, 1.586, Hδ, 3.196, 3.142, Hε, 7.247 | |  |
| G43 | 8.202 | 3.832 | 3.715 |  | |  |  | |  |
