# Supplementary figures and images for: Morintides: cargo-free chitin-binding peptides from Moringa oleifera
Source: BMC Plant Biol. 2017 Mar 31;17:68. doi: 10.1186/s12870-017-1014-6 (PMC5374622; doi:10.1186/s12870-017-1014-6)

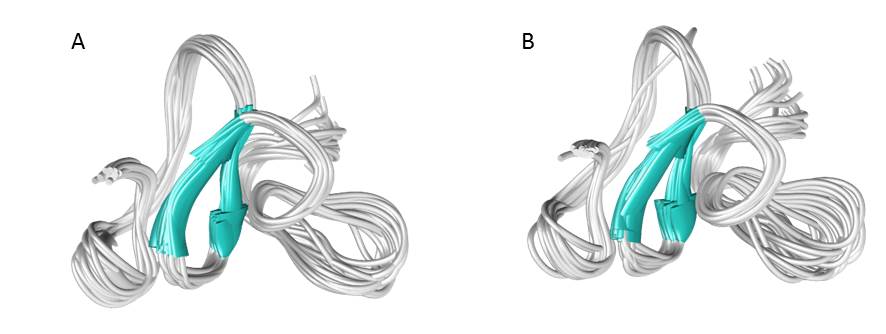

Supplement: Supplementary file 5 — The structures generated by CNSsolve 1.3 without any disulfide bonds assumed for structure calculation (A) and with the disulfide bonds combination Cys I- Cys IV, Cys II-Cys V, Cys III- Cys VI and Cys VII- Cys VII assumed for structure calculation (B) (JPG 28 kb) [file 12870_2017_1014_MOESM5_ESM.jpg]

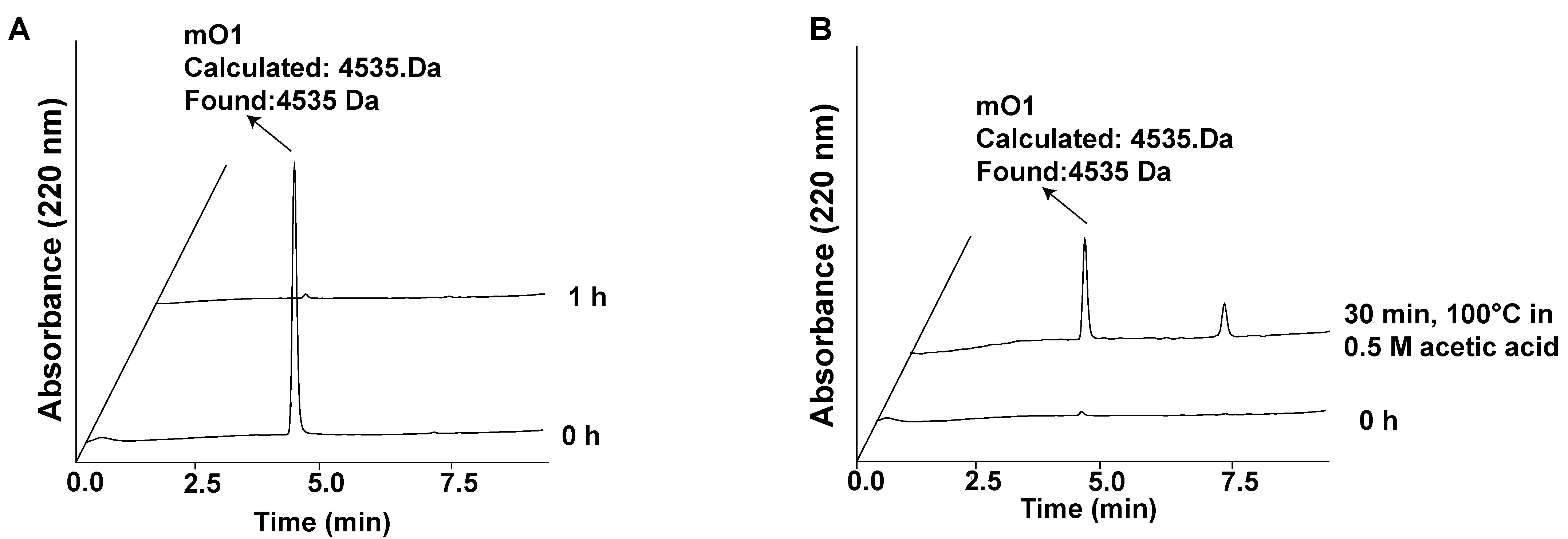

Supplement: Supplementary file 6 — Chitin-binding activity of mO1. Morintide mO1 bound to the chitin beads in 1 h and was eluted from the beads in 30 min on incubation in acidic conditions at high temperature. (JPG 357 kb) [file 12870_2017_1014_MOESM6_ESM.jpg]
