# Supplementary material for: Morintides: cargo-free chitin-binding peptides from Moringa oleifera
Source: BMC Plant Biol. 2017 Mar 31;17:68. doi: 10.1186/s12870-017-1014-6 (PMC5374622; doi:10.1186/s12870-017-1014-6)
Supplement: Supplementary file 7 — Translated nucleotide sequences of mO1 and mO2 as obtained from transcriptomic analysis. An asterisk indicates the start codon and a hyphen indicates the stop codon. (DOCX 90 kb) [file 12870_2017_1014_MOESM7_ESM.docx]

**mO1**

**atggcgaagctaagtttcttgagcttgtttcttctttgtcttgttgctactgccact**

**M* A K L S F L S L F L L C L V A T A T**

**gcccagaattgcggccgccaggcgggaaacagagcctgcgccaatcaactttgttgcagc**

**A Q N C G R Q A G N R A C A N Q L C C S**

**cagtacggcttctgtggctcaactagtgaatattgttctcgtgcaaatggctgtcagagc**

**Q Y G F C G S T S E Y C S R A N G C Q S**

**aattgcaggggcggtggaggtgccgatggtgctggcggcgaggctggtggtggaggtcct**

**N C R G G G G A D G A G G E A G G G G P**

**taa**

**-**

**mO2**

**atggcgaagctaagtttcttgagcttgtttcttctttgtcttgttgctactgccact**

**M* A K L S F L S L F L L C L V A T A T**

**gcccagaattgcggccgccaggcgggaaacagagcctgcgccaatggactttgttgcagc**

**A Q N C G R Q A G N R A C A N G L C C S**

**cagtacggcttctgtggctcaactagtgaatattgttctcgtgcaaatggctgtcagagc**

**Q Y G F C G S T S E Y C S R A N G C Q S**

**aattgcaggggcggtggaggtgccggtggtgctggcggcggggctgggggtggaagtcct**

**N C R G G G G A G G A G G G A G G G S P**

**taa**

**-**

Supplementary data S5. Translated nucleotide sequences of mO1 and mO2 as obtained from transcriptomic analysis. An asterisk indicates the start codon and a hyphen indicates the stop codon.
